# The long-term cost-effectiveness of fetal monitoring during labour: a comparison of cardiotocography complemented with ST analysis versus cardiotocography alone

**DOI:** 10.1111/j.1471-0528.2008.01935.x

**Published:** 2008-12

**Authors:** E Heintz, T-H Brodtkorb, N Nelson, L-Å Levin

**Affiliations:** a Center for Medical Technology Assessment (CMT), Linköping UniversityLinköping, Sweden; b Division of Paediatrics, Department of Clinical and Experimental Medicine, Linköping University HospitalLinköping, Sweden

**Keywords:** Cardiotocography, cerebral palsy, cost-effectiveness, fetal monitoring, ST analysis

## Abstract

**Objective:**

To assess the cost-effectiveness of the use of cardiotocography (CTG) complemented with fetal electrocardiography and ST analysis compared with the use of CTG alone in term deliveries when a decision has been made to use fetal monitoring with a scalp electrode.

**Design:**

A cost-effectiveness analysis based on a probabilistic decision model incorporating relevant strategies and lifelong outcomes.

**Setting:**

Maternity wards in Sweden.

**Population:**

Women with term fetuses after a clinical decision had been made to apply a fetal scalp electrode for internal CTG.

**Methods:**

A decision model was used to compare the costs and effects of two different treatment strategies. Baseline estimates were derived from the literature. Discounted costs and quality-adjusted life years (QALYs) were simulated over a lifetime horizon using a probabilistic model.

**Main outcome measures:**

QALYs, incremental costs, and cost per QALY gained expressed as incremental cost-effectiveness ratio (ICER).

**Results:**

The analysis found an incremental effect of 0.005 QALYs for ST analysis compared with CTG; the ST analysis strategy was also moreover associated with a €56 decrease in costs, thus dominating the CTG strategy. The probability that ST analysis is cost-effective in comparison with CTG is high, irrespective of the willingness-to-pay value for a QALY.

**Conclusions:**

Compared with CTG alone, ST analysis is cost-effective when used in term high-risk deliveries in which there is a need for internal fetal monitoring.

## Introduction

Fetal oxygen deficiency during birth can cause neurological damage, and in 0.06%^1^ of all births in Sweden, the deficiency is so severe that it leads to cerebral palsy or death. To take appropriate actions to prevent hypoxia, fetal surveillance with a scalp electrode is used in complicated deliveries. Currently, the most common method of fetal surveillance with a scalp electrode is internal cardiotocography (CTG). This method of surveillance, however, has been demonstrated to have several weaknesses and is therefore coupled with blood sampling to achieve sufficient specificity.^1^ To overcome some of these weaknesses and enhance the detection of fetuses at risk of hypoxia, a new method of combining CTG with ST analysis has been developed. This method provides an automatic analysis of the ST interval in the electrocardiogram complex of the fetus and, compared with CTG alone, is thought to rely less on subjective interpretation. Several studies comparing CTG with ST analysis have been published,^2^–^4^ and a Cochrane review^5^ concluded that use of ST analysis, compared with use of CTG alone, is associated with a reduction in cases of metabolic acidosis and operative deliveries. However, although the results of the Cochrane review indicate a positive health effect of using ST analysis, these results are limited to the intermediate outcome, namely metabolic acidosis. Hence, whether using ST analysis actually improves the long-term health outcome of the fetus has not been investigated.

As cost-effectiveness is a key criterion for decision makers when deciding what healthcare interventions should be made available in collectively funded healthcare systems,^6^ it is important to compare the estimated lifelong effects with the additional costs of adding ST analysis to the labour monitoring regime. A short-term cost-analysis^7^ by the National Institute for Clinical Excellence (NICE), not taking the potential effects on metabolic acidosis into account, compared the higher equipment costs associated with ST analysis to the costs-savings due to the lower risk of operative deliveries. They reached the conclusion that the annual net cost of using ST analysis in England would be £3.4 million but also stated that a full economic analysis is required to assess the cost-effectiveness of ST analysis.

When performing a cost-effectiveness analysis, the incremental cost between two or more treatments is divided with the incremental effect to form the incremental cost-effectiveness ratio (ICER), which represents the additional cost one has to pay to gain the effect unit of choice. When investigating the cost-effectiveness, one should capture all relevant costs and effects related to treatment choice.^8^ As the consequences of metabolic acidosis when leading to cerebral palsy are lifelong and often severe, we would argue that a cost-effectiveness analysis that only takes into account the intermediate outcome, namely metabolic acidosis, is insufficient. Rather, to capture a broader understanding of health and wellbeing, quality-adjusted life years (QALYs) is the recommended outcome to be used in economic evaluations of healthcare interventions.^8^ When calculating QALYs, the time spent in a particular health state is weighted by a quality-adjustment weight or utility, ranging between 0 (dead) and 1 (full health), representing the quality of life associated with the health state.^8^ Being a generic effect measure, the use of QALYs also enables comparisons with other treatment areas.

As no study so far has investigated the long-term effects of using ST analysis, data from different sources have to be synthesised and extrapolated to investigate the long-term outcome. Decision-analytical models offer the possibility to synthesise evidence from various sources and to extrapolate costs and effects to a relevant setting and time intervals.^9^ The purpose of our study was to, by employing a decision-analytic model, undertake a cost-effectiveness analysis comparing the long-term costs and effects (in terms of QALYs) of fetal monitoring with ST analysis in comparison with those of using CTG alone.

## Methods

To estimate the costs and effects of CTG complemented with electrocardiography (ECG) and ST analysis, a decision tree was used. By structuring the decision tree based on the results of previously reported trials, we extrapolated the costs and effects over a lifetime horizon, taking into account the probability of developing cerebral palsy and its effect on QALYs. The analysis was performed from a Swedish societal perspective, including costs related to productivity losses due to morbidity but excluding productivity losses due to mortality. Costs are expressed in Euros (€) at the 2006 price level and have been adjusted according to the Swedish Consumer Price Index.^10^ The exchange rate used in the translation of costs was 9.4 Swedish Kronor per Euro.

### Treatment strategies

The two treatment strategies analysed entailed delivering a child with the support of either CTG alone or CTG complemented with ECG and ST analysis (hereafter referred to as ‘ST analysis’). Internal CTG alone and ST analysis are used in complicated term deliveries when a decision has been made to attach a scalp electrode to the fetal head. The ST analysis strategy uses the same technology as that of CTG but is complemented with ECG and analysis of the ST waveform to rate the significance of abnormalities revealed by CTG. The fetal ECG is automatically recorded, processed, and analysed by the ST analysis monitor. When the CTG results are normal, no attention is paid to abnormalities in the ST analysis. If CTG results are abnormal or pathological, the severity of the ST event determines what intervention is the most appropriate. For a preterminal CTG result, immediate delivery is recommended.^1^ Fetal scalp blood sampling is optional for both intervention strategies.^5^

### Model structure

**Figure 1:**
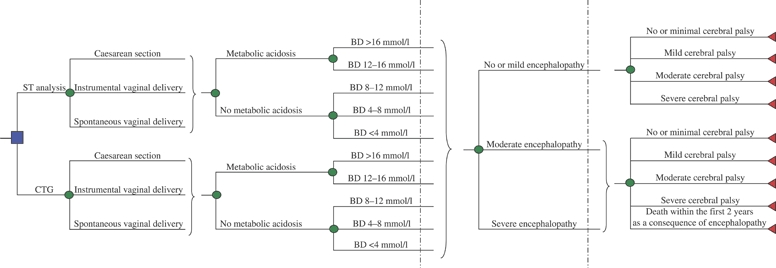
The decision-analytic model. Different modes of delivery are modelled as consequences of CTG and STAN. Regardless of the method of delivery, there is a probability that the child may manifest metabolic acidosis after birth. This probability differs between the two intervention strategies. The probability that the child develops different grades of newborn encephalopathy depends on the level of base deficit. Moderate or severe encephalopathy could lead to cerebral palsy of the different degrees of severity, but could also lead to death or development without complications. Children with no or mild encephalopathy face a risk of developing cerebral palsy due to other causes than oxygen deficiency. BD, base deficit.

The simplified model structure is shown in Figure 1. The structure of the decision tree is identical for both treatment strategies, with only the probabilities of operative deliveries and metabolic acidosis differing between the two. The long-term extrapolation of the treatment effect is equal with regard to the structure and probability of both alternatives.

In the model, a fetus being delivered faces a treatment-specific probability of being born through spontaneous vaginal, instrumental vaginal, or caesarean delivery. This results in different degrees of complications for both the fetus and the mother in the two strategies. An assumption was made that every delivery only results in the birth of one child. After birth, the child may or may not display signs of metabolic acidosis at a rate dependent on the treatment strategy. To the best of our knowledge, there is no published evidence of a direct correlation between incidence metabolic acidosis and cerebral palsy. However, we do know that on its way from an intrapartum hypoxic–ischaemic injury causing cerebral palsy, the child progresses through newborn encephalopathy.^11^ To take account of this connection, the transition from signs of metabolic acidosis to cerebral palsy was therefore modelled using levels of encephalopathy, through the child’s level of base deficit. Children were classified as having no, minor, moderate, or severe encephalopathy.^12^ Children with moderate or severe encephalopathy face an elevated risk of dying or developing cerebral palsy, although they may also develop without complications. Those classified as having no or minor encephalopathy were assumed to develop without any complications due to oxygen deficiency but may still face a risk of developing cerebral palsy due to other causes. The risk of cerebral palsy increases with the presence and severity of encephalopathy.^13^ When defining the different grades of cerebral palsy, the following classification was used: *minimal* cerebral palsy is defined as motor signs present but no functional impairment, *mild* cerebral palsy as symptoms resulting in some functional impairment, *moderate* cerebral palsy as between mild and severe (e.g. ambulant with walking frame), and *severe* cerebral palsy as ‘little purposeful voluntary action, although function may be acquired, IQ permitting’.^14^

### Probabilities

The probabilities used in the model are shown in Table 1. Three randomised controlled trials (RCTs) investigated the use of ST analysis compared with CTG^2^–^4^ alone with respect to metabolic acidosis. Analysed in a meta-analysis, ST analysis was demonstrated to have a relative risk of metabolic acidosis of 0.64 (95% CI 0.41–1.00) compared with CTG,^5^ which is used in our base-case analysis. One of the included trials, the trial by Ojala *et al.*,^4^ has been criticised for using an inappropriate method of measuring metabolic acidosis, leading to a misleading increase in the frequency of metabolic acidosis.^21^ We have therefore chosen to perform a sensitivity analysis using a meta-analysis^5^ excluding this trial. However, the RCT by Amer-Wåhlin *et al.*^2^ has been criticised for not including all observed cases of metabolic acidosis in the ST analysis arm.^22^ This criticism was answered by the research group, which argued that there were no errors in 11 of the 13 cases cited;^23^ the other 2 cases, currently being investigated, were in this study included in a sensitivity scenario.

**Table 1:** Probabilities used in the model

Probabilities	Mean value	Distribution	References
Maternal mortality after caesarean section	0.18 × 10^−3^	Beta (36, 197 745)	Hall and Bewley^15^
Maternal mortality after spontaneous and instrumental vaginal delivery	0.21 × 10^−4^	Beta (38, 1 845 919)	Hall and Bewley^15^
Pulmonary embolism after caesarean section	0.51 × 10^−3^	Beta (67, 132 342)	The National Board of Health and Welfare^17^
Deep vein thrombosis after caesarean section	0.11 × 10^−2^	Beta (141, 132 268)	The National Board of Health and Welfare^17^
Cerebral thrombosis after caesarean section	0.24 × 10^−3^	Beta (32, 132 377)	The National Board of Health and Welfare^17^
Pulmonary embolism after spontaneous and instrumental vaginal delivery	0.11 × 10^−3^	Beta (131, 1 148 627)	The National Board of Health and Welfare^17^
Deep vein thrombosis after spontaneous and instrumental vaginal delivery	0.28 × 10^−3^	Beta (326, 1 148 432)	The National Board of Health and Welfare^17^
Cerebral thrombosis after spontaneous and instrumental vaginal delivery	0.91 × 10^−4^	Beta (104, 1 148 654)	The National Board of Health and Welfare^17^
Blood transfusions after caesarean section	0.46 × 10^−2^	Beta (687, 149 621)	The National Board of Health and Welfare^17^
Blood transfusions after instrumental vaginal delivery	0.40 × 10^−2^	Beta (335, 83 578)	The National Board of Health and Welfare^17^
Blood transfusions after spontaneous vaginal delivery	0.13 × 10^−2^	Beta (1415, 1 107 807)	The National Board of Health and Welfare^17^
Sepsis after caesarean section	0.15 × 10^−2^	Beta (15, 9883)	Kankuri *et al.*^18^
Sepsis after spontaneous and instrumental vaginal delivery	0.48 × 10^−3^	Beta (16, 33 559)	Kankuri *et al.*^18^
Metabolic acidosis after CTG	0.12 × 10^−1^	Beta (49, 3967)	Neilson^5^
RR metabolic acidosis after ST analysis	0.64	Gamma (7.92, 0.08)	Neilson^5^
Caesarean section after CTG	0.86 × 10^−1^	Dirichlet (378, 619, 3404)	Neilson^5^
Instrumental vaginal delivery after CTG	0.14		Neilson^5^
Spontaneous vaginal delivery after CTG	0.77		Neilson^5^
RR caesarean section after ST analysis	0.97	Gamma (186.12, 0.01)	Neilson^5^
RR instrumental delivery after ST analysis	0.87	Gamma (269.46, 0.003)	Neilson^5^
Fetal blood sampling after CTG	0.11	Beta (490, 3911)	Neilson^5^
RR fetal blood sampling after ST analysis	0.76	Gamma (141.02, 0.01)	Neilson^5^
Base deficit of >16 mmol/l in the metabolic acidosis group	0.28	Beta (24, 63)	Östergötland County Council^19^
Base deficit of 8–12 mmol/l in the no metabolic acidosis group	0.76 × 10^−1^	Dirichlet (371, 1418, 3063)	Östergötland County Council^19^
Base deficit of 4–8 mmol/l in the no metabolic acidosis group	0.29		Östergötland County Council^19^
Base deficit of <4 mmol/l in the no metabolic acidosis group	0.63		Östergötland County Council^19^
No encephalopathy when base deficit >16 mmol/l	0.39	Dirichlet (23, 12, 17, 7)*	Low *et al.*^12^
Minor encephalopathy when base deficit >16 mmol/l	0.20		Low *et al.*^12^
Moderate encephalopathy when base deficit >16 mmol/l	0.29		Low *et al.*^12^
Severe encephalopathy when base deficit >16 mmol/l	0.12		Low *et al.*^12^
No encephalopathy when base deficit 12–16 mmol/l	0.72	Dirichlet (42, 11, 4, 1)*	Low *et al.*^12^
Minor encephalopathy when base deficit 12–16 mmol/l	0.19		Low *et al.*^12^
Moderate encephalopathy when base deficit 12–16 mmol/l	0.68 × 10^−1^		Low *et al.*^12^
Severe encephalopathy when base deficit 12–6 mmol/l	0.17 × 10^−1^		Low *et al.*^12^
No encephalopathy when base deficit 8–12 mmol/l	0.83	Dirichlet (48, 10, 0, 0)*	Low *et al.*^12^
Minor encephalopathy when base deficit 8–12 mmol/l	0.17		Low *et al.*^12^
Moderate encephalopathy when base deficit 8–12 mmol/l	0.16 × 10^−3^		Low *et al.*^12^
Severe encephalopathy when base deficit 8–12 mmol/l	0.22 × 10^−3^		Low *et al.*^12^
No encephalopathy when base deficit 4–8 mmol/l	0.95	Dirichlet (53, 1, 2, 0)*	Low *et al.*^12^
Minor encephalopathy when base deficit 4–8 mmol/l	0.18 × 10^−1^		Low *et al.*^12^
Moderate encephalopathy when base deficit 4–8 mmol/l	0.36 × 10^−1^		Low *et al.*^12^
Severe encephalopathy when base deficit 4–8 mmol/l	0.15 × 10^−3^		Low *et al.*^12^
Cerebral palsy after moderate encephalopathy	0.84 × 10^−1^	Beta (15, 163)	Badawi *et al.*^13^
Mortality the first 2 years after moderate encephalopathy	0.62 × 10^−1^	Beta (19, 287)	van de Riet *et al.*^20^
Cerebral palsy after severe encephalopathy	0.23	Beta (17, 56)	Badawi *et al.*^13^
Mortality the first 2 years after severe encephalopathy	0.67	Beta (124, 60)	van de Riet *et al.*^20^
Proportion of severe cerebral palsy in children diagnosed with moderate or severe encephalopathy	0.46	Dirichlet (15, 6, 5, 6)	Badawi *et al.*^13^
Proportion of moderate cerebral palsy in children diagnosed with moderate or severe encephalopathy	0.19		Badawi *et al.*^13^
Proportion of mild cerebral palsy in children diagnosed with moderate or severe encephalopathy	0.16		Badawi *et al.*^13^
Proportion of minimal cerebral palsy in children diagnosed with moderate or severe encephalopathy	0.19		Badawi *et al.*^13^
Cerebral palsy in children with no or mild encephalopathy	0.12 × 10^−2^	Beta (99, 82 620)	Badawi *et al.*^13^
Proportion of severe cerebral palsy in children diagnosed with no or mild encephalopathy	0.25	Dirichlet (25, 30, 32, 12)	Badawi *et al.*^13^
Proportion of moderate cerebral palsy in children diagnosed with no or mild encephalopathy	0.30		Badawi *et al.*^13^
Proportion of mild cerebral palsy in children diagnosed with no or mild encephalopathy	0.32		Badawi *et al.*^13^
Proportion of minimal cerebral palsy in children diagnosed with no or mild encephalopathy	0.12		Badawi *et al.*^13^

RR, relative risk.

* To adjust for the zero counts in some of the events in these distributions a non-informative prior of 0.01 was used in the probabilistic sensitivity analysis.

In the Cochrane review used in the analysis, metabolic acidosis was defined as cord artery blood pH of less than 7.05 and a base deficit of more than 12.0 mmol/l.^5^ To enable the transition from metabolic acidosis to encephalopathy in the model, we used estimates from a trial^12^ investigating the level of base deficit associated with encephalopathy. In this trial, the distribution of different stages of encephalopathy was presented in four different groups of children. The four groups were differentiated by the base deficit values detected in the children’s cord artery blood: children with a base deficit of 4–8, 8–12, 12–16, and >16 mmol/l. Then, using the definition of metabolic acidosis as a base deficit of more than 12.0 mmol/l, we were able to create a connection between metabolic acidosis and encephalopathy. To incorporate the distribution of base deficit levels in children born in Sweden, we used base excess data^19^ from Vrinnevi Hospital in Norrköping (base excess levels for 4939 of 5984 children born in 2004–06). Children having a base deficit of less than 4 mmol/l were not included in the study by Low *et al.*,^12^ this group did in our model not include any cases of moderate or severe encephalopathy. The probability of children being diagnosed with cerebral palsy depends on the presence and severity of newborn encephalopathy. The estimates we used for this relationship were based on a trial by Badawi *et al.*^13^ The same trial was used in determining the probability of the different degrees of cerebral palsy. The probability of death after encephalopathy, was retrieved from a meta-analysis^20^ of long-term adverse outcomes for newborns. The meta-analysis included studies following the children for a period of between 18 months and 3.5 years.

As previously mentioned, we have in our base-case analysis modelled the relationship between surveillance method and encephalopathy through metabolic acidosis. Due to the complexity of this relationship, one would preferably have long-term data investigating the direct effect of surveillance method on encephalopathy. However, the only current trial specifying the numbers of moderate and severe cases of encephalopathy, thus providing the possibility of an extrapolation to cerebral palsy, is a study by Noren *et al.*^24^ The number of participants in this study is in our opinion, however, too few to be considered to correctly represent the distribution of an unlikely event, such as encephalopathy. To eliminate some of the structural uncertainty related to modelling the relationship between surveillance and encephalopathy, we have included a sensitivity scenario where the rate of encephalopathy is based on the data from Noren *et al.*^24^

### Costs

The cost parameters used in the model can be found in Table 2. The costs of giving birth by caesarean section, instrumental vaginal delivery, and spontaneous vaginal delivery include the costs of the mode of delivery plus the costs of treating potential complications in the mother. To calculate the cost per delivery of using either ST analysis or CTG, the following formula was used:

**Table 2:** Cost parameters in the model

Cost estimates	Cost	References
Caesarean section (per delivery)	€4170	Östergötland County Council^25^
Instrumental delivery (per delivery)	€1990	Östergötland County Council^25^
Spontaneous vaginal delivery (per delivery)	€1470	Östergötland County Council^25^
Use of ST analysis (per delivery)	€53	
Use of CTG (per delivery)	€23	
Cost of purchasing ST analysis (per device)	€29,800	SBU^1^
Cost of purchasing CTG (per device)	€14,900	SBU^1^
Number of devices per obstetric unit	4	Division of Obstetrics and Gynaecology, Linköping University Hospital Personal communication
Number of deliveries per obstetric unit	2500	Personal communication
Proportion of deliveries monitored with a scalp electrode	20%	SBU1 and personal communication
Scalp electrode for ST analysis	€4.15	Neoventa Medical AB^26^
Scalp electrode for CTG	€3.94	Medexa Diagnostisk Service AB^27^
Training in ST analysis interpretation	€32,400	Östergötland County Council^25^ and personal communication
Cost per hour of a midwife	€35	Östergötland County Council^25^
Cost per hour of a physician	€71	Östergötland County Council^25^
Cost per hour of an assistant nurse	€25	Östergötland County Council^25^
Fetal scalp blood sampling (per sample)	€28	Personal communication and Triolab AB^28^
Care at SCBU metabolic acidosis	€750	County Councils of Östergötland Kalmar and Jönköping^30^
Care at SCBU mild encephalopathy	€750	County Councils of Östergötland Kalmar and Jönköping^30^
Care at SCBU moderate encephalopathy	€2110	County Councils of Östergötland Kalmar and Jönköping^30^
Care at SCBU severe encephalopathy	€2490	County Councils of Östergötland Kalmar and Jönköping^30^
Pulmonary embolism (per woman)	€3200	County Councils of Östergötland Kalmar and Jönköping^29^
Deep vein thrombosis (per woman)	€2720	Levin and Bergqvist^31^
Cerebral thrombosis (per woman)	€4040	Ghatnekar *et al.*^32^
Blood transfusion (units per woman)	€230	County Councils of Östergötland Kalmar and Jönköping^29^
Sepsis (per woman)	€4370	County Councils of Östergötland Kalmar and Jönköping^29^
Cerebral palsy 0–1 years (per year)	€58,820	Svensson *et al.*^33^ and Bouwes^34^
Cerebral palsy 2–4 years (per year)	€35,680	Svensson *et al.*^33^ and Bouwes^34^
Cerebral palsy 5–17 years (per year)	€41,480	Svensson *et al.*^33^ and Bouwes^34^
Cerebral palsy 18+ years (per year)	€70,100	Svensson *et al.*^33^ and Bouwes^34^
Production losses for individuals with severe cerebral palsy, after 20 years of age (per year)	€35,790	Statistics Sweden^35^


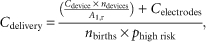


where *C*_device_ represents the cost of purchasing either CTG or ST analysis, *C*_electrode_ the annual cost of scalp electrodes, *n*_devices_ the number of devices per obstetric unit, *n*_births_ the number of deliveries per year at the obstetric unit, *p*_high risk_ the proportion of deliveries in which fetal surveillance with a scalp electrode is estimated to be used, l the life span of the equipment, r the discount factor, and *A*_l,r_ the annuity factor. The life span of the equipment was set to 7 years, and the annuity factor was 6.23.

The number of devices per obstetric unit and the number of deliveries per obstetric unit are based on data from Linköping University hospital (Table 2). To elucidate the generalisability of our results to other settings, a two-way sensitivity analysis was performed in which we simultaneously varied the number of devices per obstetric unit and the proportion of deliveries monitored with a scalp electrode. The analysis was performed for the range of two to eight devices and a proportion of monitored deliveries of 0.1–0.6.

Even though the ST analysis guidelines advocate consistent training in ST interpretation, only the training costs that occur at the introduction of ST analysis are included in our analysis. This is due to the fact that CTG also requires continuous training and that training in ST analysis interpretation is thought to affect the interpretations of CTG in a positive way.^36^ The ST analysis introduction cost was included in the cost per delivery for the ST analysis strategy and was calculated for 6 hours of training for 18 physicians and 45 midwives plus 136 physician hours and 160 midwife hours of teaching (personal communication). Included in the cost of taking a scalp blood sample were the costs of using the ABL-5 blood-gas analyser (Radiometer, Copenhagen, Denmark), the time of a doctor, a midwife, and an assistant nurse, two clinitubes, and a blood-gas syringe.^28^ Costs for care at Special Care Baby Unit (SCBU) were included for children with metabolic acidosis and different levels of encephalopathy. The average length of admission to SCBU for children with metabolic acidosis was 2.54 days (SE 0.69). The corresponding length of stay at SCBU for mild, moderate, and severe encephalopathy was 7.75 (SE 1.70), 10.75 (SE 2.32), and 12.20 (SE 4.60) days, respectively.^24^

The costs associated with the first 8 years of life for children with severe cerebral palsy were drawn from a study of the costs of the erroneous management of delivery.^33^ In this particular study, five of nine children had some degree of invalidity; one to a degree of 50–70% and four to a degree of 71–100%. As the primary diagnosis associated with severe injuries due to erroneous management during labour has been demonstrated elsewhere to be cerebral palsy,^37^ these costs are employed in our model for children with severe cerebral palsy. To determine the costs of cerebral palsy in individuals older than 8 years, figures from an American cost-of-illness study^34^ were used as weights to extrapolate the costs over a lifetime horizon. To visualise the impact of this parameter on the result, we have in addition to the probabilistic sensitivity analysis included two sensitivity scenarios in which we have augmented and decreased the costs by 50%. Individuals with severe cerebral palsy were assumed to be unable to work. The annual costs of productivity losses were drawn from official data collected by Statistics Sweden.^35^ In the model, the average age of beginning a first job was set to 20 years and productivity losses were calculated for ages 20–64 years. The effect of including these productivity losses in our analysis was tested in a sensitivity analysis.

### Life expectancy and QALYs

To the best of the authors’ knowledge, no complete age-specific standard mortality statistics are available for the definitions of cerebral palsy used in the model. However, evidence of survival time for the definitions used in the model does exist for the time interval of 25–40 years of age, dependent on the degree of cerebral palsy.^14^ The findings of this study were incorporated into our model by employing a parametric time-to-event survival model with a Weibull distribution.^38^ The results of the Weibull regression indicated a decreasing hazard of death with respect to age as the ancillary gamma parameter declined below 1 (i.e. −0.2, SE 0.08); the constant in the regression was −3.51 (SE 0.21). Using appropriate formulas, the fitted hazard function was transformed to yearly probabilities of death. The nature of the Weibull regression will lead to a continuing decrease in the probability of death, not accounting for the natural increase late in life. The probability of death was therefore only based on the regression as long as it remained above the standard mortality.^39^ As the probability of death for the children with encephalopathy already is included in the model, the first 2 years of the curves were excluded for these children to avoid double counting of the death risk. Due to the uncertainty in this extrapolation of death risk, a sensitivity scenario was analysed based on a time horizon of only 25 years.

Health outcomes in the analysis were expressed as QALYs. The QALY weights used in the model are shown in Table 3. How these are used in the calculation of QALYs is illustrated by an example of a QALY for a 40-year-old individual with moderate cerebral palsy:

**Table 3:** QALY weights

	QALY weights	References
Severe cerebral palsy	0.04	Rosenbaum *et al.*^40^ and Bax *et al.*^41^
Moderate cerebral palsy	0.45	Rosenbaum *et al.*^40^ and Bax *et al.*^41^
Mild cerebral palsy	0.84	Rosenbaum *et al.*^40^ and Bax *et al.*^41^
General population <30	0.90	Burstrom *et al.*^42^
General population 30	0.88	Burstrom *et al.*^42^
General population 40	0.87	Burstrom *et al.*^42^
General population 50	0.85	Burstrom *et al.*^42^
General population 60	0.82	Burstrom *et al.*^42^
General population 70	0.78	Burstrom *et al.*^42^
General population 80+	0.69	Burstrom *et al.*^42^


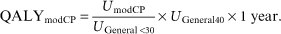


As QALY weights for individuals diagnosed with mild, moderate, and severe cerebral palsy are only available for adolescents,^40^ the *U*_modCP_, representing the QALY weight for children with moderate cerebral palsy, was divided by *U*_General <30_, representing the QALY weight for the corresponding age group in the general population. This generated an age- and severity-adjusted QALY factor that could be multiplied by the utility of the general population with the corresponding age, *U*_General40_. In this way, we incorporated a decrease in QALY weights with respect to increasing age equal to that of the general population.^42^ As cerebral palsy has been internationally defined as a nonprogressive impairment,^43^ we incorporated no additional decrease in the QALY weights with respect to age for individuals with cerebral palsy compared with the general population.

The QALY weights used in our analysis were classified according to the Gross Motor Function Classification System (GMFCS). As the degrees of cerebral palsy used in our model were not based on the GMFCS levels, we assumed that the values for level I, levels II and III, and levels IV and V correspond to how Blair *et al.*^14^ defined mild, moderate, and severe cerebral palsy, respectively.^41^

### Analysis

According to recent guidelines, the model was analysed using second-order Monte-Carlo simulation to reflect uncertainty in the model inputs.^44^ In this approach, values are drawn randomly from defined probability distributions in the model, and costs and QALYs are established for both strategies. This process is repeated 5000 times to provide 5000 estimates of the ICER, reflecting the uncertainty in the outcome. The uncertainty surrounding the estimated ICER also reflects the uncertainty surrounding the decision to employ a ST analysis strategy rather than a CTG strategy. The proportion of the 5000 simulations resulting in an ICER below the threshold value at which decision makers are willing to pay for a QALY represents the probability of ST analysis being cost-effective.^45^ As there is no single established true willingness-to-pay for a QALY, this probability can be presented for a range of willingness-to-pay values in so-called cost-effectiveness acceptability curves (CEACs), allowing the decision maker to select the willingness-to-pay for a specific treatment strategy and then assess the probability that the strategy is cost-effective. Costs and QALYs were discounted by 3% per annum, in line with recent guidelines.^46^

## Results

The results of the base-case analysis (Table 4) showed that the use of ST analysis results in a gain in QALYs at a lower cost and therefore dominates CTG. The lower overall cost of using the ST analysis strategy, despite the higher initial costs of ST analysis, is partly due to the avoidance of costs related to cerebral palsy. However, even in the time period until just after delivery, ST analysis is cost saving in comparison with CTG alone (Table 5). This is explained by the lower rate of complicated deliveries, cases of metabolic acidosis, and cases of encephalopathy in the ST analysis arm.

**Table 4:** Comparison of costs and effects

Method	Costs	Incremental costs	Effectiveness (QALYs)	Incremental effectiveness	ICER
ST analysis	€3349	−56	27.1636	0.0054	Dominating
CTG	€3405		27.1582		

**Table 5:** Contribution to total costs (calculated for one delivery)

Costs per delivery	CTG	ST analysis	Incremental cost
Equipment cost	€23	€53*	€30
Delivery method + care at SCBU	€2163	€2118	−€45
Severe cerebral palsy	€983	€949	−€34
Production losses	€236	€229	−€7
Total cost	€3405	€3349	−€56

* Introductional training in ST analysis interpretation is included in the equipment cost.

**Figure 2:**
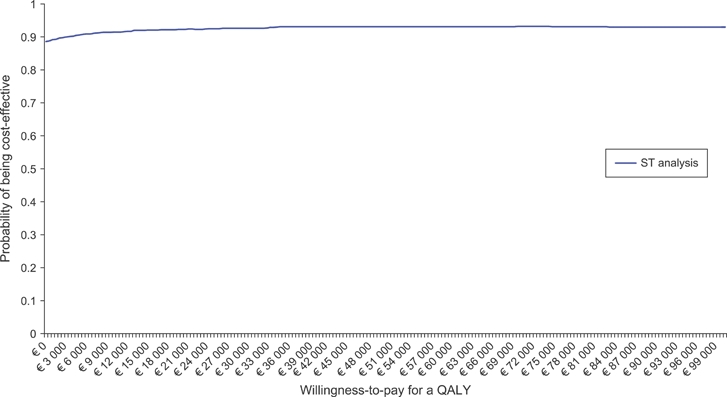
The cost-effectiveness acceptability curve.

The probabilistic sensitivity analysis demonstrated that the probability of ST analysis being cost-effective was more than 0.9 for all willingness-to-pay values over €9000, as shown in the CEAC presented in Figure 2.

### Sensitivity analysis

None of the results from the sensitivity scenarios led to a different conclusion concerning the cost-effectiveness of ST analysis (Table 6).

**Table 6:** Results of the sensitivity scenarios

	Method	Costs	Incremental costs	Effectiveness (QALYs)	Incremental Effectiveness	ICER
Excluding data for metabolic acidosis in the trial by Ojala *et al.*^4^	ST analysis	€3308	−€115	27.1660	0.0092	Dominating
	CTG	€3423		27.1568		
25-year time horizon	ST analysis	€2800	−€37	16.4671	0.0033	Dominating
	CTG	€2837		16.4638		
Including two extra cases of metabolic acidosis in the ST analysis arm	ST analysis	€3357	−€49	27.1630	0.0048	Dominating
	CTG	€3405		27.1582		
Excluding production losses for individuals with severe cerebral palsy	ST analysis	€3120	−€48	27.1636	0.0054	Dominating
	CTG	€3168		27.1582		
Using data on encephalopathy from the Swedish RCT^24^	ST analysis	€2363	−€228	27.1682	0.0291	Dominating
	CTG	€2592		27.1973		
Augmenting the costs of severe cerebral palsy with 50%	ST analysis	€3823	−€73	27.1636	0.0054	Dominating
	CTG	€3896		27.1582		
Decreasing the costs of severe cerebral palsy with 50%	ST analysis	€2874	−€40	27.1636	0.0054	Dominating
	CTG	€2914		27.1582		

Except for the scenario with eight devices and a proportion of 0.1 deliveries monitored with a scalp electrode (31.25 deliveries per equipment and year), all other scenarios varying the number of devices and proportion of deliveries monitored with either CTG or ST analysis, resulted in ST analysis still dominating the CTG strategy. For the scenario with eight devices and 0.1 deliveries monitored with a scalp electrode, the costs were, however, so low that ST analysis would still be considered cost-effective, yielding a incremental cost of €11 and an incremental effect of 0.0054 QALYs, that is an ICER of €2105 per QALY.

## Discussion

The purpose of this study was to evaluate the cost-effectiveness of ST analysis compared with that of CTG alone. Our study has shown that in a comparison of the two strategies, ST analysis strategy is the cost-effective alternative. When including both the immediate direct treatment costs at the hospital and the lifetime costs for individuals with severe cerebral palsy, the results indicate that ST analysis is a cost-saving method compared with CTG. However, also when considering only the treatment costs associated with the immediate care at the hospital, our study shows that ST analysis is cost saving, which means that the additional cost of using the ST analysis device instead of CTG is more than outweighed by the costs saved by avoiding caesarean sections, instrumental vaginal deliveries, cases of metabolic acidosis, and cases of encephalopathy. This result is in contradiction to previous results in the cost-analysis performed by NICE,^7^ which indicates that the cost of purchasing the ST analysis equipment is higher than the potential cost savings from reduced operative deliveries. Their analysis did, however, not include costs for care of children with metabolic acidosis and neonatal encephalopathy. Neither did it include an equipment cost for the CTG alone strategy. The ST analysis equipment consists of both the CTG and the additional ST analysis and is, when implemented, thought to replace the alternative of CTG alone. We therefore argue that the equipment cost of ST analysis must be compared with that of CTG alone. The costs of introductory training was in our analysis, however, only included for the ST analysis strategy and have been calculated for the circumstances at Linköping University Hospital. So has the equipment number per obstetric unit and the number of deliveries per obstetric unit, both used in the calculations of the costs of equipment per delivery. Using these data in the calculations of the equipment, cost per delivery corresponds to using a number of approximately 125 deliveries per device and year. The conclusion regarding the cost-effectiveness of ST analysis did, however, not change when in a scenario analysis varying the number of devices per obstetric unit and the proportion of deliveries monitored with a scalp electrode. This therefore warrants a generalisability of our results to other settings.

That ST analysis, compared with CTG alone, helped reduce the number of cases of metabolic acidosis was previously demonstrated in a Cochrane review.^5^ Our analysis demonstrates that the use of ST analysis also results in a gain in QALYs, when taking into account the effects of cerebral palsy and death. As cerebral palsy in our model was defined as a nonprogressive impairment (i.e. the physical state was assumed not to change with age only because of the disease), the QALY weights we used in our analysis decrease with age based on the changes of quality-of-life data in the general population. We do not know how an individual with cerebral palsy perceives that the quality of life changes as he or she grows older. The difference in mobility between people with and without cerebral palsy is, for example, believed to increase with age during childhood and adolescence, but an individual with cerebral palsy may, however, also cope with the disease with time. Concerning the lifetime costs of cerebral palsy, no cost data were available for the costs of care for individuals with mild and moderate cerebral palsy. Including such costs would have strengthened the cost-effectiveness of ST analysis further, as according to our model, there are fewer cases of cerebral palsy after using ST analysis than after using CTG alone. Production losses were included for both the relatives of children with severe cerebral palsy and the children themselves as adults.^33^,^35^ The sensitivity scenario excluding the costs of production losses for the children with cerebral palsy did not yield results changing the conclusion that ST analysis should be considered cost-effective.

The link between metabolic acidosis and cerebral palsy in our model was established using previous research into this relationship. This was carried out to model the path followed by a child affected by oxygen deficiency severe enough to cause cerebral palsy. The concept of extrapolating costs and outcomes by modelling a longer time period than that for which available trial data exist might seem clinically doubtful. When evaluating cost-effectiveness, however, the appropriate time horizon should be the time over which the costs and effects of the alternatives might differ.^8^ The time the children remain alive is often the only relevant time horizon, even if the children are never followed for that period in trials. In this study, evaluating different methods of fetal monitoring, the information gap between what has been observed in clinical trials and the expected future effects on health and costs literally includes a whole lifetime. The extrapolation to cerebral palsy and death was in our model performed in two steps involving various data sources. To validate the model, one could compare the probability of developing cerebral palsy according to this model with outcomes in clinical studies of the subject. Himmelmann *et al.*^47^ concluded that the mean prevalence of term cerebral palsy in Sweden was 11.1 per 10 000 live births. The prevalence of cerebral palsy in our model was higher, 21.30 and 21.83 of 10 000 in the ST analysis and CTG arms, respectively, a difference that could perhaps be explained by the use of Australian and Canadian studies when determining the probabilities of encephalopathy and cerebral palsy (the overall rate of term cerebral palsy in the Australian study was 15.8 per 10 000).^13^ We also compared the prevalence of encephalopathy in the model with those established in the literature. Badawi *et al.*^48^ have estimated the general birth prevalence of moderate or severe encephalopathy to be 3.8 per 1000 term live births. In comparison with this number, the rates of encephalopathy in our model are high (12.1 and 12.8 per 1000 in the CTG and ST analysis arms, respectively). However, the difference seems to be mainly due to an augmented number of encephalopathy due to causes other than metabolic acidosis, and this affects the cost-effectiveness of ST analysis negatively. According to Low *et al.*,^12^ the prevalence of metabolic acidosis plus moderate or severe encephalopathy is approximately 3 per 1000. In our model, the same number is 2.1 and 1.4 per 1000 births in the CTG arm and ST analysis arm, respectively, which is reasonable as we have lower rates of metabolic acidosis. The sensitivity scenario investigating the use of the data on encephalopathy from Noren *et al.*^24^ showed that ST analysis was even more cost-effective when basing our model on these direct observations.

The validity of our results is of course dependent on the validity of the studies upon which it has been based. We therefore find it important to mention that the results of two of the included RCTs have been questioned.^21^,^22^ The criticism of Ojala *et al.*^4^ concerned the method used to measure metabolic acidosis. The inclusion of this trial in the base-case analysis reduced the difference between the two strategies in cases of metabolic acidosis and operative deliveries. However, ST analysis was still the dominant intervention, and excluding the results of this trial from the sensitivity analysis meant that using ST analysis resulted in even higher gains of QALY at lower costs. Amer-Wåhlin *et al.*^2^ has on the other hand been criticised for excluding cases of metabolic acidosis from the ST analysis arm. When in a sensitivity scenario including two additional cases of metabolic acidosis in the ST analysis arm the differences in QALYs and costs between the two strategies were slightly reduced, but the ST analysis strategy was still dominating the CTG strategy.

As pointed out earlier in the discussion, our analysis is not without limitations. With regard to the decision on whether ST analysis should be implemented, results from a long-term trial would of course be desirable. However, despite lack of such evidence, decisions concerning the method of fetal surveillance will still have to be made. It is important to remember that the alternative to using the current approach of synthesising current best available evidence is implicit decision-making with little or no information about the rationale for decisions. By employing a probabilistic decision-analytic model, we have been able to calculate the cost-effectiveness of ST analysis and take the uncertainty of the current evidence into account. Although some of the assumptions made in the analysis may seem dubious to the reader, all sensitivity scenarios have shown that the results are robust with regard to these assumptions. Based on our results, a decision maker may make an informed decision while waiting for the results from a long-term trail. As the first study fully assessing the cost-effectiveness of ST analysis, it contributes valuable information, both concerning the cost-effectiveness ratio and the uncertainty concerning the decision.

## Conclusions

According to the results of our evaluation, the use of ST analysis results both in a gain in QALYs and in lower costs when compared with CTG alone. Thus, ST analysis is the cost-effective alternative when used in complicated term deliveries in which a decision has been made to monitor the fetus with a scalp electrode.

## Disclosure of interest

The authors retained full control over the manuscript’s content and the decision of submission.

## Contribution to authorship

All authors contributed to the conception and design of the study, interpretation of results, and critical revision of the manuscript. E.H. collected the data. E.H. and T.H.B. developed the model, performed the analysis and completed the initial draft of the manuscript.

## Details of ethics approval

No application to ethical committee was necessary.

## Funding

The study was partly financed by an unrestricted grant from Neoventa Medical AB.
